# Novel causes and consequences of overtraining syndrome: the EROS-DISRUPTORS study

**DOI:** 10.1186/s13102-019-0132-x

**Published:** 2019-09-18

**Authors:** Flavio A. Cadegiani, Claudio E. Kater

**Affiliations:** Adrenal and Hypertension Unit, Division of Endocrinology and Metabolism, Department of Medicine, Federal University of São Paulo (Unifesp/EPM), Rua Pedro de Toledo 781 – 13th floor, São Paulo, SP 04039-032 Brazil

**Keywords:** Athletes, Conditioning, Endocrine and metabolic responses on overtraining syndrome (EROS) study, Performance, Overtraining syndrome

## Abstract

**Background:**

Hormonal physiology in athletes, dysfunctional paths leading to overtraining syndrome (OTS), and clinical and biochemical behaviors that are independently modified by the presence of OTS remain unclear. Although multiple markers of OTS have recently been identified, the independent influence of OTS on hormones and metabolism have not been assessed. Hence, the objective of the present study was to uncover the previously unrecognized independent predictors of OTS and understand how OTS independently modifies the behaviors of clinical and biochemical parameters.

**Methods:**

In a total of 39 athletes (OTS-affected athletes (OTS) = 14 and healthy athletes (ATL) = 25), we performed two clusters of statistical analyses using the full data of the Endocrine and Metabolic Responses on Overtraining Syndrome (EROS) study, in a total of 117 markers. We first used logistic regression to analyze five modifiable parameters (carbohydrate, protein, and overall caloric intake, sleep quality, and concurrent cognitive effort) as potential additional independent risk factors for OTS, and OTS as the outcome. We then used multivariate linear regression to analyze OTS as the independent variable and 38 dependent variables. Training patterns were found to be similar between OTS and ATL, and therefore excessive training was not a risk, and consequently not a predictor, for OTS.

**Results:**

Each of the three dietary patterns (daily carbohydrate, daily protein, and daily overall calorie intake) were found to be the independent triggers of OTS, while sleeping, social, and training characteristics depended on other factors to induce OTS. Once triggered, OTS independently induced multiple changes, including reductions of cortisol, late growth hormone and adrenocorticotropic hormone responses to stimulations, testosterone-to-estradiol ratio, neutrophils, neutrophil-to-lymphocyte ratio, vigor levels, hydration status, and muscle mass, while increase of tension levels and visceral fat.

**Conclusions:**

OTS can be independently triggered by eating patterns, regardless of training patterns, while the occurrence of OTS reduced late hormonal responses and the testosterone-to-estradiol ratio, worsened mood, and affected the immunology panel. These novel findings may explain underperformance, which is the key characteristic of OTS.

## Introduction

Overtraining syndrome (OTS) is characterized by a prolonged and unexplained decrease in sports performance usually associated with severe psychological manifestations [1]. It is caused by an imbalance among training, social, sleep, and eating patterns, which leads to metabolic, endocrine, and biochemical changes [2–7] related to a long-term shortage of energy and mechanisms of repair [1, 3, 6, 8]. Despite the name “overtraining syndrome”, referring to excessive exercise training, other modifiable factors may trigger OTS [3, 4, 9]. Although excessive training disrupts physiological processes leading to OTS, whether and how eating, social, and sleep patterns disrupt adaptive changes in athletes remains uncertain. Challenges and issues in the methodology used for the assessment of OTS include the failure to identify relevant biomarkers and pathophysiology.

Given the gap in knowledge about OTS triggers, we conducted the Endocrine and Metabolic Responses to Overtraining Syndrome (EROS) study [9–13], to enhance our understanding of the pathophysiology and biomarkers of OTS and assessment tools for the early recognition, and prevention of OTS. The EROS study [9–13] compared OTS-affected athletes (OTS group), healthy athletes (ATL group), and non-physically active controls on 117 parameters. The identification of more than 45 new biomarkers and mechanisms of OTS showed that disruptions in the adaptive changes of athletes led to a breakdown in multiple pathways, thereby leading to OTS. However, despite the multiple novel findings in OTS, we were unable to identify independent predictors of OTS and parameters that were independently disrupted by the presence of OTS without a joint analysis of all the results of the EROS study [9–13] the use of more complex statistical tools.

The objective of the present study was to understand whether and which factors independently trigger OTS, aside from excessive training, and how OTS independently leads to changes in behaviors in multiple clinical, metabolic, and biochemical markers. The uncovering of these mechanisms in the present study was intended to improve our understanding of the etiology and consequences of OTS, and to develop additional tools for the prevention and precise diagnosis of OTS.

## Methods

For the present analysis, we performed a comprehensive joint statistical analysis of data from five of the arms of the EROS study, including four of primary findings: 1.) the EROS-HPA axis, in which we evaluated the hypothalamic-pituitary-adrenal axis hormonal responses in athletes [9]; 2.) the EROS-STRESS, in which we evaluated the prolactin and growth hormone (GH) responses to an exercise-independent stimulation test – the insulin tolerance test (ITT) – as well as the glucose behavior during this test [10]; 3.) the EROS-PROFILE, in which we evaluated eating, psychological, sleeping, and social patterns [11]; 4.) the EROS-BASAL, in which we evaluated: basal hormones; inflammatory; immune; and muscular parameters [12]; and 5.) the additional EROS-HIFT arm, in which we evaluated specific characteristics of healthy and OTS-affected HIFT (including CrossFit) athletes [3].

Full descriptions of the materials and methods (i.e.*,* the selection of participants and study procedures), results of the statistical analyses of data, and their respective discussions are available in these five of the arms of the present study [9–13], as well as in a depository (https://osf.io/bhpq9), which also has the raw data of the results of each participant.

### Subject selection

We recruited participants through calls for participation in social media and group messages, and invitations to sports coaches. Prior to interview, each candidate self-reported sex, age, body mass index (BMI), and whether he intended to participate as a healthy athlete, clinically suspected for OTS, or healthy sedentary. Aiming homogenous groups, we specified criteria for all groups, of OTS-affected athletes (OTS group), healthy athletes (ATL group), and non-physically active controls (NPAC group) including sex (male), age (18–50 years old), BMI (20–29.9 kg/m2 for sedentary and 20–32.9 kg/m^2^ for athletes), absence of known hormonal, metabolic, inflammatory, or psychiatric disorders, non-current or recent use of drugs or hormones. In order to avoid false athletes, for the two groups of athletes we required a minimum amount of training per week (> 300 min and > four times a week), intensity of training (at least moderate-to-intense, according to their sport coaches), time since started non-stop training (> 6 months). To avoid misdiagnosis of OTS, for athletes suspected of OTS, we required a sports-coach certified reduction of at least 10% of previous performance, or a loss of > 20% in time to fatigue, increased sense of effort for a same training intensity and volume, persistent fatigue that lasted > 2 weeks, unresponsive to resting, and lack of use of confounding drugs or hormones, and presence of confounding diseases. For all candidates that fulfilled criteria for any of the two groups, we performed hormones and basic biochemical profile and avoided those who presented alterations in any of the tested parameters.

### Identification of independent triggers and consequences of OTS

**Fig. 1:**
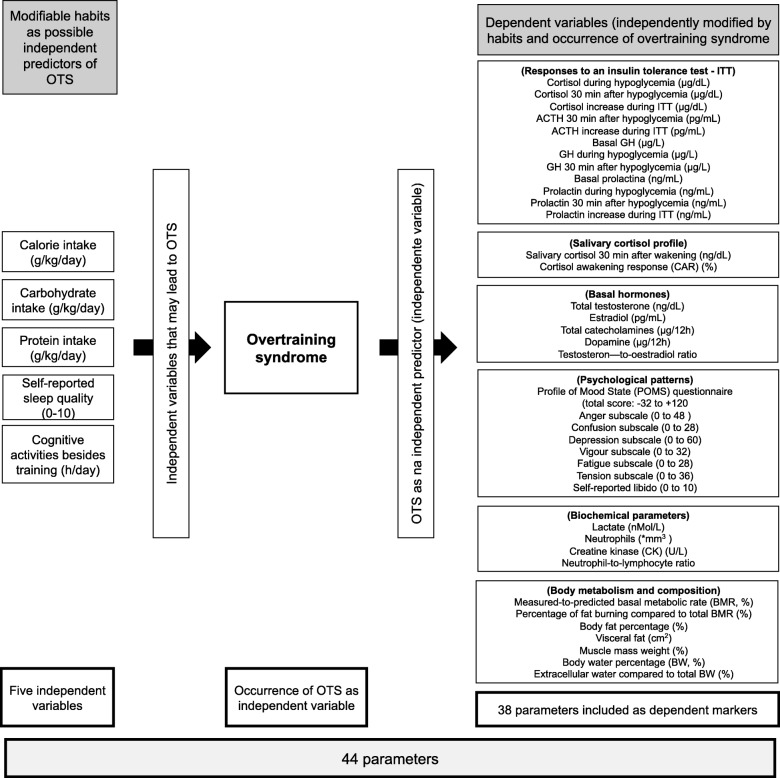
Variables included in the present analysis

In the present study, we performed a joint multivariate and logistic regression analyses for the identification of independent triggers and consequences of OTS, among those parameters that were suitable for the diagnosis or as characteristic of OTS, and significantly different between OTS and ATL. From the 117 evaluated parameters in the EROS study [9–13], 31 were non-diagnostic, not useful, or unsubstantiated; nine were qualitative (yes vs no), three had missing data in > 5% of participants, and 27 had similar levels (Table 1). Hence, we selected 44 hormonal responses to stimulation tests, basal and accumulated hormonal levels, social and psychological aspects, specific eating patterns, and body metabolism and composition parameters, among which 38 were variables dependent of five modifiable variables (eating, sleeping, and social patterns) plus the presence of OTS as an additional variable, in a total of 44 variables (38 dependent and 6 independent variables) with two groups of athletes: the OTS and ATL groups (*N* = 39; OTS = 12 and ATL = 25) (Fig. 1). Additional analyses of the excluded parameters n were also performed, but were not included The variability of the biochemical markers measured in all arms of the EROS study were as low as 3.5 and 3.0% for inter- and intra-assay coefficients, respectively.

**Table 1:** Markers included in the present analysis, among those evaluated by the EROS study

Study/Tests	Markers *Total number of markers: 117* *(Included:45)*	Whether included or excluded (and if excluded, why)
EROS-HPA axis	*Total number of markers: 20*	*Included markers: 7*
Basal ACTH and cortisol and their response to an insulin tolerance test (ITT)	1. Basal cortisol (μg/dL) 2. Cortisol during hypoglycemia (μg/dL) 3. Cortisol 30 min after hypoglycemia (μg/dL) 4. Cortisol increase during ITT (μg/dL) 5. Basal ACTH (pg/mL) 6. ACTH during hypoglycemia (pg/mL) 7. ACTH 30 min after hypoglycemia (pg/mL) 8. ACTH increase during ITT (pg/mL) 9 Basal ACTH/cortisol ratio 10. ACTH/cortisol ratio during hypoglycemia 11. ACTH/cortisol ratio 30 min after hypoglycemia	Similar levels between OTS and ATL INCLUDED INCLUDED INCLUDED Similar levels between OTS and ATL Similar levels between OTS and ATL INCLUDED INCLUDED Unsubstantiated marker Unsubstantiated marker Unsubstantiated marker (although different between OTS and ATL)
Cortisol response to a *cosyntropin* stimulation test (CST)	12. Cortisol at 30 min after synthetic ACTH shot (μg/dL) 13. Cortisol at 60 min after synthetic ACTH shot (μg/dL) 14. Difference between basal cortisol on day 1 (CST) and day 3 (ITT) (%)	Similar levels between OTS and ATL Similar levels between OTS and ATL Not diagnostic or helpful
Salivary cortisol rhythm (SCR)	15. Salivary cortisol at awakening (ng/dL) 16. Salivary cortisol 30 min after wakening (ng/dL) 17. Salivary cortisol at 4 PM (ng/dL) 18. Salivary cortisol at 11 PM (ng/dL) 19. Cortisol awakening response (CAR) (%) 20. Difference between 8 AM and 4 PM salivary cortisol (%)	Similar levels between OTS and ATL INCLUDED Similar levels between OTS and ATL Similar levels between OTS and ATL INCLUDED Not diagnostic or helpful
EROS-STRESS	*Total number of evaluated markers: 12*	*Included markers: 7*
GH and Prolactin response to an ITT	1. Basal (GH) (μg/L) 2. GH during hypoglycaemia (μg/L) 3. GH 30 min after hypoglycaemia (μg/L) 4. Basal prolactin (ng/mL) 5. Prolactin during hypoglycaemia (ng/mL) 6. Prolactin 30 min after hypoglycaemia (ng/mL) 7. Prolactin increase during ITT (ng/mL) 8. Basal serum glucose (mg/dL) 9 Serum glucose during hypoglycemia (mg/dL) 10. Capillary glucose during hypoglycemia (mg/dL) 11. Adrenergic symptoms during hypoglicemia (0–10) 12. Neuroglycopenic symptoms during hypoglicemia (0–10)	INCLUDED INCLUDED INCLUDED INCLUDED INCLUDED INCLUDED INCLUDED Not diagnostic or helpful Not diagnostic or helpful Not diagnostic or helpful Not diagnostic or helpful (although different between OTS and ATL) Not diagnostic or helpful
EROS-BASAL	*(total number of evaluated markers: 32)*	*Included markers: 9*
Hormonal markers	1. Total testosterone (ng/dL) 2. Estradiol (pg/mL) 3. IGF-1 (pg/mL) 4. TSH (μUI/mL) 5. Free T3 (pg/mL) 6. Total catecholamines (μg/12 h) 7. Total metanephrines (μg/12 h) 8. Noradrenaline (μg/12 h) 9. Epinephrine (μg/12 h) 10. Dopamine (μg/12 h) 11. Metanephrine (μg/12 h) 12. Normetanephrine (μg/12 h) 13. Catecholamine-to-metanephrine ratio	INCLUDED INCLUDED Similar levels between OTS and ATL Similar levels between OTS and ATL Similar levels between OTS and ATL INCLUDED Similar levels between OTS and ATL Similar levels between OTS and ATL Similar levels between OTS and ATL INCLUDED Similar levels between OTS and ATL Similar levels between OTS and ATL Not diagnostic or helpful (although diferente between OTS and ATL)
Biochemical markers	14. Erythrocyte sedimentation rate (ESR, mm/h) 15. Hematocrit (%) 16. C-reactive protein (CRP, mg/dL) 17. Lactate (nMol/L) 18. Vitamin B12 (pg/mL) 19. Ferritin (ng/mL) 20. Neutrophils (*mm^3^) 21. Lymphocyte (*mm^3^) 22. Eosinophils (*mm^3^) 23. Creatine kinase (CK, U/L) 24. Medium corpuscular volume (MCV) 25. Platelets (10^3^/mm) 26. Low density lipoprotein cholesterol (LDLc) (mg/dL) 27. High density lipoprotein cholesterol (HDLc) (mg/dL) 28. Tryglicerides (mg/dL)	Similar levels between OTS and ATL Similar levels between OTS and ATL Similar levels between OTS and ATL INCLUDED Similar levels between OTS and ATL Similar levels between OTS and ATL INCLUDED Similar levels between OTS and ATL Similar levels between OTS and ATL INCLUDED Not diagnostic or helpful Not diagnostic or helpful Data missed in > 5% of participants Data missed in > 5% of participants Data missed in > 5% of participants
Ratios	29. Testosterone-to-oestradiol ratio 30. Testosterone-to-cortisol ratio 31. Neutrophil-to-lymphocyte ratio 32. Platelet-to-lymphocyte ratios	INCLUDED Similar levels between OTS and ATL INCLUDED Similar levels between OTS and ATL
EROS-PROFILE	*(total number of evaluated markers: 53)*	*Included markers: 21*
Nutritional patterns (7-day diet record, prior and during OTS)	1. Calorie intake (kcal/kg/day) 2. Carbohydrate intake (g/kg/day) 3. % calories from carbohydrate (%) 4. Protein intake (g/kg/day) 5. % calories from protein (%) 6. Fat intake (g/kg/day) 7. % calories from fat (%) 8. Carbohydrate intake > 3 g/kg/day (Y/N) 9. Daily whey protein consumption (Y/N) 10. Followed a diet plan (Y/N) 11. Post-workout carbohydrate intake > 0.5 g/kg (Y/N)	INCLUDED (as a modifiable habit^a^) INCLUDED (as a modifiable habit^a^) Intrinsically linked to other parameters INCLUDED (as a modifiable habit^a^) Intrinsically linked to other parameters Similar levels between OTS and ATL Intrinsically linked to other parameters Qualitative marker Qualitative marker Qualitative marker Qualitative marker
Psychological patterns (during OTS)	12. Profile of Mood State (POMS) questionnaire (total score: −32 to + 120) 13. Anger subscale (0 to 48) 14. Confusion subscale (0 to 28) 15. Depression subscale (0 to 60) 16. Vigour subscale (0 to 32) 17. Fatigue subscale (0 to 28) 18. Tension subscale (0 to 36) 19. How do you fell today? (0–10) 20. Have you been sick in the last two weeks? (Y/N)? 21. How was your last training session compared to the projected goals? (Extremely easy to extremely hard) 22. How do your muscles feel? (Nothing at all to extremely painful) 23. How friendly do you feel today? (0–6) 24. How worthless do you feel today? (0–6) 25. How miserable do you feel today? (0–6) 26. How helpful do you feel today? (0–6) 27. How bad-tempered do you feel today? (0–6) 28. How unworthy do you feel today? (0–6) 29. How peeved do you feel today? (0–6) 30. How cheerful do you feel today? (0–6) 31. How sad do you feel today? (0–6) 32. Number of hours of activities besides professional training (h/day)	INCLUDED INCLUDED INCLUDED INCLUDED INCLUDED INCLUDED INCLUDED Not diagnostic or helpful (although different between OTS and ATL) Qualitative marker Not diagnostic or helpful (although different between OTS and ATL) Not diagnostic or helpful (although different between OTS and ATL) Not diagnostic or helpful Not diagnostic or helpful Not diagnostic or helpful Not diagnostic or helpful (although different between OTS and ATL) Not diagnostic or helpful Not diagnostic or helpful Not diagnostic or helpful Not diagnostic or helpful (although different between OTS and ATL) Not diagnostic or helpful INCLUDED (as a modifiable habit^a^)
Social patterns (during OTS)	33. Duration of night sleep (h) 34. Self-reported sleep quality (0–10) 35. Self-reported libido (0–10) 36. Initial imnsonia (Y/N) 37. Terminal imnsonia (Y/N) 38. More than two wake-ups during sleep (Y/N) 39. Work and/or study (Y/N) 40. Libido during resting periods / vacations (0–10)	Similar levels between OTS and ATL INCLUDED (as a modifiable habit^a^) INCLUDED Qualitative marker Qualitative marker Qualitative marker Qualitative marker Not diagnostic or helpful
Body metabolism analysis (indirect calorimetry)	41. Measured-to-predicted basal metabolic rate (BMR, %) 42. Percentage of fat burning compared to total BMR (%)	INCLUDED INCLUDED
Body composition (Bod Pod, InBody770 and 3D body scanner)	43. Body fat percentage (%) 44. Visceral fat (cm^2^) 45. Muscle mass weight (%) 46. Body water percentage (BW, %) 47. Extracellular water compared to total BW (%) 48. Body weight (kg) 49.Chest to waist circumference 50. Waist circumference (cm) 51. Chest circumference (cm) 52. Biceps circumference (cm) 53. Hip circumference (cm)	INCLUDED INCLUDED INCLUDED INCLUDED INCLUDED Not diagnostic or helpful Not diagnostic or helpful Not diagnostic or helpful Not diagnostic or helpful Not diagnostic or helpful Not diagnostic or helpful

*OTS* Athletes affected by overtraining syndrome, *ATL*, Healthy athletes

^a^For statistical purposes, modifiable factors were considered as independent variables, from which the dependent variables were statistically evaluated

### Statistical analysis

Of 44 markers, we used logistic regression to analyze five independent variables and one dependent variable in order to identify independent triggers of OTS. Five modifiable habits, including caloric, protein, and carbohydrate intake, sleep quality, and the number of hours spent working or studying were the independent predictors, and the presence of OTS was the outcome. We used multivariate linear regression with six independent variables, including the five modifiable variables plus the presence of OTS (as the sixth independent variable), and the markers among the 38 remaining variables that were significantly different between the OTS and ATL groups, as the dependent variables. The purpose was to elucidate the role of OTS as an independent modifier of body composition and metabolism, and biochemical, hormonal, and psychological markers.

All statistical analyses were performed using SAS 9.4 (SAS Institute, Inc., Cary, NC). Logistic regression analyses were performed using the five independent variables and binominal codes for the presence or absence of OTS as the dependent variable; 35.9% of the participants had OTS (*p* < 0.05). Given the context of the present study and its main objective, the number of participants in the present study was found to be sufficient for the number of variables and outcomes for the present logistic regression analyses. We used multivariate linear regression with the backward variable selection method (removal criterion = *p* > 0.01) to analyze the significance of the contributions of the 44 variables. This process was performed until none of the predictors met the removal criterion. The standardized residual variables of the last performed model were examined for normality, homoscedasticity, and multicollinearity. The tolerance index of the remaining variables in the last model was ≥0.40^3^. A *p-*value < 0.05 for all analyses was considered statistically significant.

## Results

In the EROS study, from the 87 athletes suspected of OTS and 46 healthy athletes that were initially recruited, 14 were selected for the OTS group (83.9% of the initial candidates for OTS were excluded due to exclusion of the actual diagnosis of OTS) and 25 for the ATL group. The baseline characteristics of age (OTS = 30.6 years and ATL = 32.7 years) and body mass index (BMI) (OTS = 26.7 kg/m^2^ and ATL = 24.9 kg/m^2^), and the training patterns including training intensity (OTS = 8.79 and ATL = 8.76, on a scale from zero to ten), frequency (OTS = 5.36 days and ATL = 5.46 days) and period (OTS = 574.3 min and ATL = 550.0 min a week), and time since started training non-stop were statistically similar between OTS and ATL. All 14 participants selected for the OTS group had true and naturally occurring presence of OTS, not functional or non-functional overreaching, as all athletes had a verified decrement of > 10% of previous sports performance and fatigue that were prolonged (average duration of fatigue and decreased performance = 44.3 ± 23.0 days), and none had fully recovered by the time of the study. Supplementary information regarding the selection process and baseline characteristics have been previously published [3–7].

The independent triggers of OTS are presented in Table 2. The variables that were independently modified by the presence of OTS, their degree of association, and the estimation equation for each of these variables are presented in Table 3.

**Table 2:** Independent triggers of OTS

*Scenario*	Independent variables included	Results (* = positive for independent risk factors and triggers)	Interpretation
*Scenario* 1 – All modifiable variables	CHO, PROT, CAL, WORK, SLEEP	PERFECT SEPARATION	Together, modifiable patterns were able to explain all cases of OTS in the athletes studied.
*Scenario* 2 – All modifiable variables, except WORK	CHO, PROT, CAL, SLEEP	PERFECT SEPARATION	Dietary patterns together with sleep quality were also able to fully explain all cases of OTS in the studied population of athletes.
*Scenario* 3 – All modifiable variables, except CAL	CHO, PROT, WORK, SLEEP	CHO: *p* = 0.036 OR/CL = 1.61 (1.03–2.50) PROT: *p* = 0.029 OR/CL = 16.7 (1.34–208.1) WORK: p = n/s SLEEP: *p* = 0.069 OR/CL = 2.19 (0.94–5.09)	When daily caloric intake is not accounted, not all cases of OTS may be justified. However, in this scenario both CHO and PROT were shown to be independent triggers of OTS.
*Scenario* 4 – Without specification of each macronutrient	CAL, WORK, SLEEP	CAL: *p* = 0.004 OR/CL = 1.13 (1.04–1.23) WORK: p = n/s SLEEP: p = n/s	When each macronutrient intake is not specified, not all cases of OTS may be justified. However, in this scenario CAL was enough to independent etiology of OTS.
*Scenario* 5 – Only dietary patterns	CHO, PROT, CAL	CHO: p = n/s PROT: *p* = 0.066 OR/CL = 25.85 (0.81–825.3) CAL: *p* = 0.045 OR/CL = 1.27 (1.01–1.61)	When only dietary patterns are evaluated, we cannot explain all cases of OTS in the studied population. However, in this scenario, overall caloric intake, but not each macronutrient, was able to

CHO Daily carbohydrate intake (g/kg/day), *PROT* Daily protein intake (g/kg/day), *CAL* Mean daily caloric intake (kcal/kg/day), *WORK* Average number of working or studying hours a day, besides training sessions (h/day), *SLEEP* Self-reported sleep quality (0–10), *OTS* Overtraining syndrome, *OR* Odds ratio, *CL* 95% Confidence Limits, *p* Level of significance, *n/s* non-significant (*p* > 0.1)

**Table 3:** Clinical and biochemical behaviors independently modified by overtraining syndrome (OTS)

Parameters modified by the presence of OTS	*p* of the influence of OTS ^a^	Level of influence of the presence of OTS ^a^ (Adjusted R-Square)	Other variables that may also influence	Equation for the estimation of the parameter level in male athletes
Late ACTH response to an ITT (30’after hypoglycaemia) (pg/mL)	0.002	19.9%	*none*	*n/a*
Late cortisol response (30’after hypoglycaemia) (μg/dL)	0.0005	26.1%	*none*	Cortisol (μg/dL) = 17.86–3.81(if OTS)
Cortisol response to an ITT (μg/dL)	0.002	22.0%	*none*	*n/a*
Late GH response (30’after hypoglycaemia) (μg/L)	0.001	23.0%	*none*	*n/a*
Testosterone-to-oestadiol ratio (T/E)	0.0002	30.7%	*none*	T/E = 14.1 + 12.9 (if OTS)
POMS vigour subscale	< 0.0001	83.6%	Sleep quality	POMS vigour subscale = 3.7 + 1.15x(sleep quality) – 11.96(if OTS)
POMS fatigue subscale	< 0.0001	85.7%	Sleep quality	POMS fatigue subscale = 24.5–0.9 x(sleep quality) + 15.3(if OTS)
POMS tension subscale	< 0.0001	42.8%	*none*	Not able to be estimated
Visceral fat (cm^2^)	0.002	38.2%	Protein and overall calorie intake	Visceral fat = 47.4–11.9x(protein intake) + 1.3x(calorie intake) + 45.1(if OTS)
Muscle mass (%)	0.028	33.7%	Protein intake	Muscle mass = 47.84 + 1.42x(protein intake) – 3.47(if OTS)
Body water (%)	0.001	50.5%	Protein and overall calorie intake	Body water = 60.75 + 1.69x(protein intake) – 0.12x(calorie intake) - 5.77(if OTS)
Neutrophils (/mm^3^)	0.015	13.8%	Calorie intake	Neutrophils = 4210–60.7x(calorie intake) + 154.4x(CHO intake) -1724(if OTS)
Neutrophil-to-lymphocyte ratio	0.015	13.6%	*none*	Ratio = 2.00–1.32(if OTS)

*CHO* Carbohydrate, *ITT* Insulin tolerant test, *POMS* Profile of mood states, *BMR* Basal metabolic rate, *T/E* Testosterone-to-oestradiol, *OTS* Overtraining syndrome’, *n/a* non applicables (non-normal distribution)

Calorie intake = kcal/kg/day, CHO intake = g(CHO)/kg/day; protein intake = g(protein)/kg/day; extra activities = working and/or studying hours besides training, sleep quality = self-reported sleep quality (0 to 10)

^a^Other minor influences may also reflect the p-value and the level of influence

When analyzed together, at least one factor between low carbohydrate, low protein intake, low overall caloric intake, and poor sleep quality was present in 100% of the study’s cases of OTS. Carbohydrate intake was found to be an independent trigger of OTS when it was analyzed together with sleep and social patterns, with an odds ratio (OR) = 1.61, [confidence limits (CL) = 1.03–2.50] for the risk of developing OTS, while its ability to induce OTS was lost without the concurrent analysis of sleep quality.

Conversely, protein intake was shown to independently induce OTS without the concurrent analysis of any of the other possible triggers in all scenarios. Likewise, overall caloric intake independently induced OTS, irrespective of the proportions of macronutrients, indicating that if caloric intake (but no carbohydrate or protein intake), work hours, and sleep quality had been analyzed together as the three modifiable habits, caloric intake would have been the only independent trigger (*p* = 0.004; OR = 1.13 [CL = 1.04–1.23]) between these three variables. In contrast, excessive work and poor sleep quality, each failed to induce OTS independently, regardless of the combinations of predictors.

Among the parameters that were statistically different between the OTS and ATL groups, the presence of OTS independently blunted late responses of adrenocorticotropic hormone (ACTH), cortisol, and growth hormone (GH) to an insulin tolerance test (ITT). This accounted for 20, 26, and 23% of their responses, respectively, while later prolactin, and early ACTH, cortisol, GH, and prolactin responses were unaffected by OTS.

With respect to the basal hormones, OTS reduced the testosterone-to-estradiol (T:E) ratio by 43%, while it did not modulate total testosterone, estradiol, or any of the other hormones. Conversely, the basic immunology panel, including neutrophils, lymphocytes, and the neutrophil-to-lymphocyte ratio were influenced by the occurrence of OTS, although at lower degrees of association, and only when combined with other triggers.

OTS also affected tension, fatigue, and vigor levels when evaluated through the Profile of Mood States (POMS) questionnaire, accounting for 43, 84, and 86% of their levels, respectively. While OTS did not affect any aspect of body metabolism (ratio between measured and expected basal metabolic rate (BMR) and percentage of fat oxidation), it independently led to reductions in muscle mass and body water content to 34 and 51%, respectively, and an increase in visceral fat to 38%. While visceral fat was increased in OTS, overall body fat was unchanged by the presence of OTS.

## Discussion

Despite the identification of multiple markers among clinical, metabolic, and biochemical parameters in OTS athletes in the EROS study [9–13], we were unable to identify specific patterns or a standard group of biomarkers for OTS, as each affected athlete exhibited a unique combination of altered markers. In the absence of a unique accurate biomarker for the diagnosis of OTS, we observed that combinations of markers that were significantly different between the OTS and ATL groups could potentially lead to a precise diagnosis of OTS, with an accuracy of 100% to distinguish OTS athletes from healthy athletes. Despite the successful ability to identify affected athletes, we were unable to identify independent triggers of OTS, as our previous analyses did not identify the influences on or causes of OTS at the individual level. Moreover, the analyses did not enhance our understanding of how each of the modifiable patterns and the occurrence of OTS independently induced changes in the behaviors of multiple clinical and biochemical markers (i.e., inherent changes caused by each modifiable factor, and changes that were independently modified by OTS itself, not by its triggers).

The *post-hoc* use of multivariate linear regression and logistic regression, which were not used in the previous EROS studies on OTS [9–13], identified the factors that independently led to OTS, and the parameters that were inherently modulated by the presence of OTS. To understand the correlations between OTS and its triggers, and OTS and its consequences, we investigated which modifiable factors could be independent causes of OTS, (i.e., whether a specific modifiable factor was solely responsible for the occurrence of some cases of OTS). We also examined which parameters might be independently modified by the presence of OTS, irrespective of other characteristics (i.e., even with the same caloric, protein, and carbohydrate intake, the same sleep quality and duration, the same amount of additional sports-related activity, and the same training intensity, volume, frequency, and duration). Our aim was to identify whether and how the mere presence of OTS modified the behaviors of the tested parameters. Specifically, among the intrinsic mechanisms of OTS, which were inherently responsible for at least some of the dysfunctional changes found in OTS, as consequences, not causes, of OTS. The dysfunctional adaptations in the clinical and biochemical aspects induced by the modifiable factors, plus the changes in these parameters were inherently due the occurrence of OTS, which was triggered by the same modifiable factors that also led to changes in the behaviors of multiple parameters. In a negative synergistic process, in which dysfunctions were enhanced by the concurrent insufficient carbohydrate, protein, and/or caloric intake, or poor sleep quality, and the presence of OTS, they were also induced by these factors, whereby both changeable factors and the presence of OTS increased the dysfunctions induced by both factors. This vicious cycle probably plays an important role in the challenging recovery process of OTS, as these factors can have a “snowball effect,” which precludes the healing process.

The use of both healthy and OTS-affected athletes for the logistic regression analyses was important to predict behavior patterns prior to OTS, as the development of OTS may be understood as a process on a continuum (i.e., the end of an unresolved mixture of attempts to adapt to chronic energy depletion and the mechanisms underlying a recovery-deprived environment) [1–3, 8, 9, 14, 15]. The significant differences in clinical, hormonal, metabolic, psychological, and biochemical behaviors between the ATL and OTS groups, when all variables were perfectly adjusted for baseline characteristics, and training, eating, social, and sleep patterns, supported the conclusion that these changes in behaviors were inherently due to the presence of OTS, as the occurrence of OTS was shown to independently increase tension levels and blunt vigor levels, while may independently enhance fatigue, as a sort of a vicious cycle, since fatigue is also one of the features of OTS. Given the data generated in the present study, the relationship between physiological and pathological behavior patterns suggest these are early signs of future dysfunction (OTS), and therefore, should be used as a warning signal in clinical practice. These differentiations and the pathophysiological paths have provided us with a more comprehensive understanding of OTS.

### Independent triggers of overtraining syndrome: beyond excessive training

Excessive training has traditionally been viewed as the major cause of unexplained reductions in sports performance, and therefore, referred to as “overtraining syndrome.” However, given advances in knowledge about the importance of periodized training, excessive training is now considered a minor factor in the development of OTS.

Unexpectedly, the incidence of OTS did not decrease with the improvements in training patterns, nor did it show a paradoxical increase; perhaps this finding is due to the growing number of athletes. Given this context, despite the clear existence of OTS triggers other than excessive training, these findings had not been reported prior to the EROS study.

As all the training patterns were similar between the healthy and OTS-affected athletes in the EROS study, excessive training was not found to be a trigger for all of the affected athletes, which allowed us to identify novel etiologies of OTS. In the EROS-PROFILE arm [11], dietary (i.e., carbohydrate, protein, and total caloric intake), social (i.e., the number of hours spent working or studying), and sleep (e.g., sleep quality) patterns were found to have a role in the development of OTS, as these parameters were significantly different between OTS and ATL group. However, whether any of these triggers were independent or dependent upon a combination of triggers was not examined in this arm of the EROS study.

The combination of OTS triggers identified in the EROS study using logistic regression explained all cases of OTS among the participants (i.e., the combination was shown to be “the perfect predictor”). Even without the independent variable of number of hours worked, the combination of dietary and sleep patterns was still found in all of the OTS cases. Conversely, dietary patterns alone, or the combination of two of the three dietary characteristics with other factors did not explain OTS in any of the affected athletes. Therefore, all dietary patterns plus sleep quality need to be assessed in order to identify athletes at risk for OTS. However, not all possible triggers are needed to develop OTS. In addition, it is important to mention that a very high odds ratio is likely to be a statistical overestimation of an association of different variables when one variable is the sole predictor of an outcome (in this case, OTS) without controlling for other variables.

Carbohydrate, protein, or overall caloric intake may each, independently disrupt physiological responses to a sport; hence, OTS can be induced without the presence of any of the other risk factors. Noteworthy, OTS is more likely to occur after changes in eating, sleeping and/or social patterns. In clinical practice, dietary characteristics should be assessed prior to other triggers, and whenever they do not indicate the presence of OTS, sleep and social patterns should be investigated. However, there is not a specific threshold for each activity or habit, as each the influence of them will highly depend on the combination with other potential triggers of OTS.

### Overtraining syndrome as an independent predictor of clinical, metabolic, and biochemical behaviors

Our findings help provide novel tools to identify athletes at risk for developing OTS and for its prevention; this approach is more efficient than the management of the challenges associated with recovery from OTS. Specific outcomes related to these findings are described below.

Although early hormonal responses to the ITT were predicted independently and positively by carbohydrate intake, the presence of OTS predicted their late responses (except for prolactin). Indeed, the commencement of a physical activity at maximum capacity for a short period, which is represented by early responses to stimulation and unaffected by OTS, is not typically observed in athletes with OTS. Conversely, reduced time-to-fatigue, a hallmark of OTS, can be explained by the blunted late hormonal responses independently predicted by the presence of OTS. This indicates an inability to maintain hormonal responses for longer periods in the presence of OTS, which probably explains the reduced pace and impaired performance of athletes during training sessions and competitions.

Among the basal hormones, the T:E ratio [12], but not any single hormone, was disrupted by the presence of OTS. The T:E ratio was found to be a better predictor of metabolic and psychological parameters than testosterone or estradiol alone [12, 16–22], as the benefits of increased estradiol in males were apparent only with a concurrent increase in testosterone [18, 19, 22]. Testosterone alone did not have the same benefits as the simultaneous increase of both testosterone and estradiol [16–18]. The benefits of an increase in estradiol accompanied by an increase in testosterone contrasted with the harmful effects of increased estradiol without an increase in testosterone, which is explained by whether the underlying mechanisms that raise estradiol levels are physiological or pathological. Estradiol physiologically increases in response to increased testosterone, and therefore, both levels are higher; however, a rise in estradiol may be a pathological increase due to an exacerbation of aromatase activity, which is present in metabolic and inflammatory dysfunctions, such as obesity and diabetes. The best way to discern whether an estradiol increase has a physiological or pathological cause, using a single marker, is through the T:E ratio, which is unaffected by physiological situations and reduced by aromatase exacerbations, as in the case of an estradiol increase, leading to a testosterone decrease. A reduced T:E ratio might be additional evidence that OTS, regardless of its triggers, induces an anti-anabolic, dysfunctional, and energy-saving environment to reduce testosterone as a protective mechanism against energy expenditure and anabolic activity by its conversion into estradiol by the enzyme aromatase. However, the underlying mechanisms that lead to a reduced T:E ratio in OTS are unknown. The EROS study showed that a T:E ratio should be greater than 13.7:1.0 (for total testosterone and estradiol are expressed in ng/mL and pg/dL, respectively) [12].

The basic immunology panel was also independently affected by the presence of OTS, which supports the theory of involvement of the immune system in the pathophysiology of OTS. Although altered immunology panels (i.e., altered when compared with healthy athletes, but similar to those of non-athletes) may be linked to blunted hormonal responses to stress [23, 24], the immunology panel and the hormonal responses to stimulation did not exhibit linear correlations or predictions, at least for the immunologic markers analyzed in the present study: neutrophils, lymphocytes, and the neutrophil-to-lymphocyte ratio. Other mechanisms, such as an environment with chronic stressors leading to OTS may directly predict leukocyte composition [25].

The relative dehydration, the decrease in muscle mass, and the increase in visceral fat, which were independently induced by OTS, may have been caused by the multiple dysfunctions associated with this syndrome. The highly oxidative and inflammatory environment that occurs in OTS might have caused increased visceral fat without a concurrent increase in overall body fat.

The impaired mood induced by OTS may contribute to the severe psychological effects of OTS, which are sometimes not fully recoverable. Interestingly, although depression has been reported to be one of the outcomes of OTS [1, 3, 6], this parameter was not predicted by OTS. The harmful changes in both body composition and mood also may have roles in previously unexplained decreases in performance, which is the key and sine-quo-non characteristic of OTS.

Overall, the findings of the various arms of the EROS study led to a new understanding of the underlying mechanisms, risk factors, and diagnosis of OTS, including its pathophysiology, as a mix of failures in the conditioning processes that are typically observed in athletes. Our findings also showed that excessive training results from a combination of different triggers, including insufficient caloric intake, excessive physical and concurrent cognitive effort, and poor sleep quality, instead of the traditional theory centered on overtraining.

We hypothesized that any type of disruption in eating, sleep, social, or training patterns could lead to a spread of dysfunctional reactions through multiple pathways, as a “domino effect,” leading to aberrant changes in hormonal, muscular, immunologic, metabolic, and/or physical behaviors, and ultimately, leading to OTS, if not promptly addressed. Although not demonstrated herein, psychological dysfunctions could also play a role in the pathogenesis of OTS. The key premise of this hypothesis is that any imbalance among psychological, sleep, eating, training, or social characteristics (not only excessive training) may lead to OTS; this has been reported extensively in the different arms of the EROS study [9–13].

Usually, a complex and unique combination of different types of dysfunctions lead to OTS, suggesting that each affected athlete should have an individual combination of parameters that are positive for OTS. Hence, OTS can be diagnosed only by using multiple indices, which was supported by all the study’s cases of OTS, which may be explained only if all possible triggers are assessed, as performed in this study using logistic regression. We suggest that further studies on OTS should always assess at least eating, training, psychological, and social patterns. Although we did not evaluate different sports, the importance of each aspect as part of the pathophysiology of OTS may vary according to the type of sport practiced. However, regardless of the type of sport, the most important aspects of OTS impairment are the rapid reduction in pace during long training sessions and reduced time-to-fatigue, which are both typically found in athletes with OTS. The failure to achieve prolonged optimization of hormonal responses in OTS is likely responsible for athletes’ decreased performance and reduced pace.

**Fig. 2:**
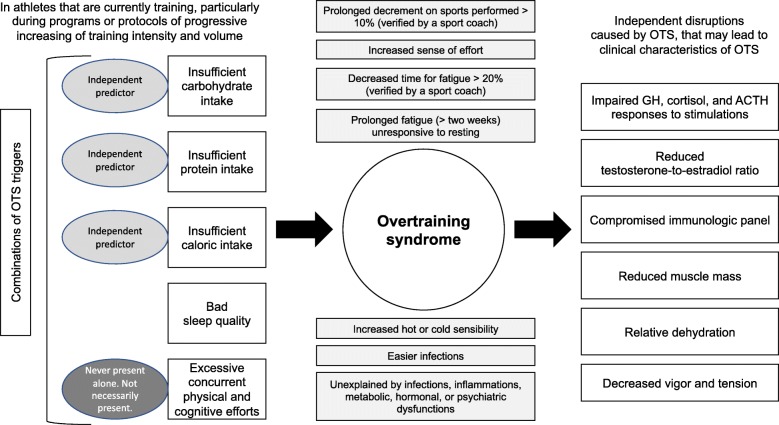
Summary of the predictions of Overtraining syndrome (OTS) and its implications

The summary of the independent predictors of OTS and its disruptions on clinical and biochemical behaviors is illustrated in Fig. 2.

### Limitations

The EROS study only evaluated male athletes that practiced either both endurance and strength modalities, or sports that demand both endurance and strength efforts. As the present study did not analyze athletes of endurance, strength, or explosive (“stop-and-go” sports, such as ball games) modalities, it is uncertain whether the findings on OTS can be replicated to these athletes, as well as female athletes. Further studies with larger samples of athletes are crucial to confirm whether our data are reproducible; longitudinal studies are needed because the present study’s design precludes drawing conclusions from the sequence of events in response to interventions in modifiable patterns, including training, eating, and social aspects.

## Conclusions

We identified that insufficient protein, carbohydrate, or overall caloric intake may trigger OTS without the presence of other triggers, while the combination of dietary patterns and sleep quality may explain all cases of OTS. Once triggered, OTS leads to a failure to achieve optimization of prolonged hormonal responses, which may explain reduced time-to-fatigue and decreased performance in long-duration sports. It also causes a reduction of the T/E ratio, paradoxical **worsened** of body composition and metabolism, failure to benefit from immunologic adaptations observed in healthy athletes, worsening vigor, fatigue, and tension, and decreased muscle mass and hydration. Worse body composition and impaired mood may also have roles in the deterioration of athletes’ performance, the hallmark of OTS.

## Data Availability

The raw data of the present study is available at https://osf.io/bhpq9/.

## Abbreviations

ACTH: Adrenocorticotropic hormone
ATL: Healthy athletes
CL: Confidence limits
EROS: Endocrine and Metabolic Responses on Overtraining Syndrome
GH: Growth hormone
ITT: Insulin tolerance test
OR: Odds ratio
OTS: Overtraining syndrome
POMS: Profile of Mood States
T:E: testosterone-to-estradiol
